# Supplementary material for: The Chimeric Antigen Receptor T Cell Target Claudin 6 Is a Marker for Early Organ-Specific Epithelial Progenitors and Is Expressed in Some Pediatric Solid Tumor Entities
Source: Cancers (Basel). 2025 Mar 7;17(6):920. doi: 10.3390/cancers17060920 (PMC11940025; doi:10.3390/cancers17060920)
Supplement: Supplementary file 1 [file cancers-17-00920-s001.zip › cancers-3476729-supplementary.pdf]

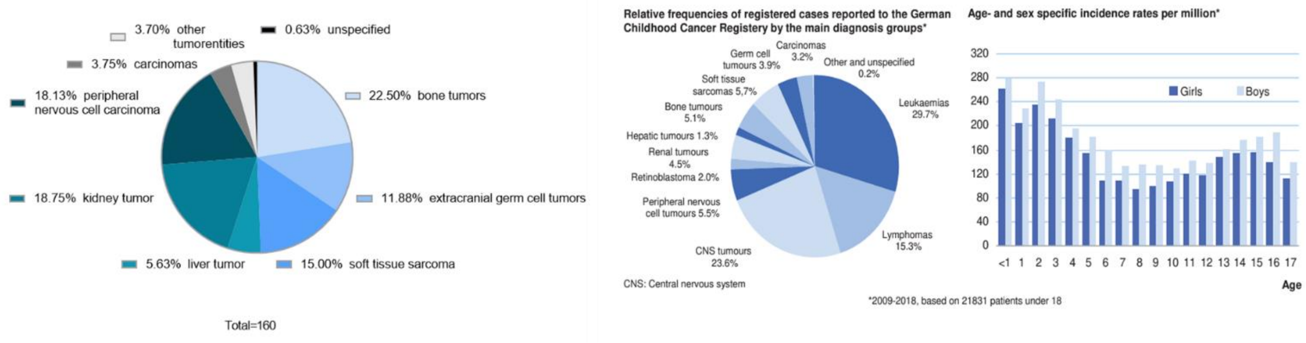

Supplementary Figure S1. Cohort. Initial cohort of extracranial tumor tissues (left) and distribution of pediatric tumor entities in Germany (right).

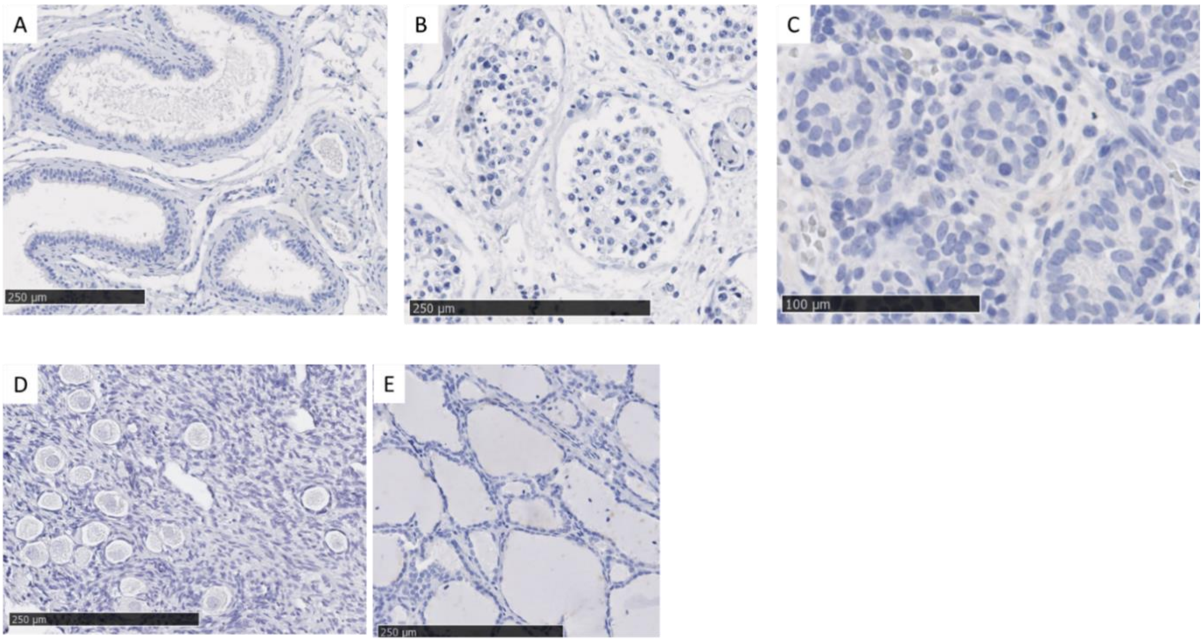

Supplementary Figure S2. CLDN6 negative tissues. IHC analysis showing CLDN6 negativity of the epithelium in epididymis (A, 17y), testis (B 17y; C 4y), ovary (D, 2 y), thyroid (E 15y). y=years.

Supplementary Table S1. Patients. Features of GCT, DSRCT, MRT and nephroblastoma analysed by qRT-PCR and/or IHC. n.d: not determined. Within the germ cell tumors, “mixed” referred to the presence of dysgerminoma and yolk sac elements.

ID	Entity	sex	age	sample	Subtype	localisation	preoperative therapy	classification	outcome	Risk	qRT-PCR value	IHC (% positive cells membrane) (intensity)	IHC (% positive cells nucleus or cytoplasm) (intensity)
1	extracranial germ cell tumor	f	14Y0M	primary	teratoma (mature)	adnexa	n	G0 Gonzalez	follow up		3,34E+03		50% (2,5+)
2	extracranial germ cell tumor	f	1Y 3M	primary	teratoma (mature)	coccyx	n	G0 Gonzalez	follow up		7,82E+03	n.d.	
3	extracranial germ cell tumor	f	9Y0M	primary	teratoma (mature)	mediastinal	n	G0 Gonzalez	follow up		1,90E+03	n.d.	
4	extracranial germ cell tumor	f	10Y2M	primary	teratoma (mature)	ovary	n	G0 Gonzalez	follow up		6,39E+03	n.d.	
5	extracranial germ cell tumor	m	0Y5M	primary	teratoma (immature)	testicle	n	G1 Gonzalez	follow up		2,49E+04	n.d.	
6	extracranial germ cell tumor	f	0Y0M	primary	teratoma (mature)	sacrococcygeal	n	G0 Gonzalez	follow up		1,62E+03	n.d.	
7	extracranial germ cell tumor	f	0Y0M	metastasis	teratoma (immature)	sacrococcygeal	n	G1 Gonzalez	follow up		1,56E+04	n.d.	
8	extracranial germ cell tumor	f	1Y0M	primary	yolk sac tumor	vaginal	n		follow up		2,88E+06	95/90% (2,5+/3+)	
9	extracranial germ cell tumor	f	5Y0M	Relapse	yolk sac tumor	pelvis minor	n		† 1 Y after diagnosis		1,42E+05	80% (2,5+)	
10	extracranial germ cell tumor	f	9Y3M	primary	yolk sac tumor	ovary	y		follow up		1,58E+03	n.d.	
11	extracranial germ cell tumor	f	8Y2M	primary	mixed	uterus	n		follow up		4,00E+05	95% (2,5+)	
11	extracranial germ cell tumor	f	8Y2M	primary	mixed	uterus	n		follow up		6,27E+04	n.d.	
11	extracranial germ cell tumor	f	8Y3M	primary	mixed	uterus	y		follow up		6,74E+03	n.d.	
12	extracranial germ cell tumor	f	11Y1M	n.d.	dysgerminoma	ovary	n.d.	n.d.	n.d.		n.d.	95% (3+)	
13	soft tissue sarcoma	m	16Y5M	primary	DSRCT	pelvis minor	y		under therapy		5,79E+05	100% (3+)	
14	soft tissue sarcoma	f	2Y6M	primary	Malignant rhabdoid tumor	cervical	y	n.d.	follow up		1,36E+03		50% nuclear (1+)
14	soft tissue sarcoma	f	2Y	metastasis	Malignant rhabdoid tumor	lymph node	y	n.d.	follow up		3,13E+04	n.d.	
15	soft tissue sarcoma	f	2Y1M	metastasis	Malignant rhabdoid tumor	intracranial	y	n.d.	† 2M after diagnosis		2,62E+03	n.d.	
15	soft tissue sarcoma	f	1Y11M	primary	Malignant rhabdoid tumor	liver	n	n.d.	† 2M after diagnosis		5,82E+03	0% (0+)	
15	soft tissue sarcoma	f	1Y11M	primary	Malignant rhabdoid tumor	liver	n	n.d.	† 2M after diagnosis		5,82E+03	n.d.	
16	soft tissue sarcoma	m	15Y7M	primary	Malignant rhabdoid tumor	pelvis minor	n	n.d.	follow up		n.d.	0% (0+)	
16	soft tissue sarcoma	m	16Y	primary	Malignant rhabdoid tumor	bladder	y	n.d.	follow up		n.d.	0% (0+)	
17	soft tissue sarcoma	n.d.	n.d	n.d.	Malignant rhabdoid tumor	n.d.	n.d.	n.d.	n.d.		n.d.	0% (0+)	
18	kidney tumor	f	10Y2M	metastasis	nephroblastoma	lymph node	y	diffuse anaplasia Stadium 3	† 2Y after diagnosis	High Risk	1,77E+05	5% (3+)	
18	kidney tumor	f	10Y2M	metastasis	nephroblastoma	thoracal	y	diffuse anaplasia Stadium 3	† 2Y after diagnosis	High Risk	2,49E+04	n.d.	
19	kidney tumor	f	2Y8M	primary	nephroblastoma	kidney	y	focal anaplasia	follow up	Intermediate	4,58E+03	50% (3+)	
20	kidney tumor	f	5Y1M	primary	nephroblastoma	kidney	y	regressive type	follow up	Intermediate	5,53E+03	n.d.	
21	kidney tumor	m	2Y10M	primary	nephroblastoma	kidney	y	regressive type	follow up	Intermediate	3,53E+04	n.d.	
22	kidney tumor	f	12Y5M	primary	nephroblastoma	kidney	y	regressive type	follow up	Intermediate	1,66E+03	n.d.	
23	kidney tumor	f	0Y8M	primary	nephroblastoma	kidney bilateral	y	regressive type	follow up	Intermediate	2,23E+03	n.d.	
24	kidney tumor	f	5Y 10M	Relapse	nephroblastoma	kidney	y	regressive type	follow up	Intermediate	n.d.	0,5% (1+)	
24	kidney tumor	f	6Y 9M	Relapse	nephroblastoma	pelvis	y	blastemic type	follow up	Intermediate	n.d.	1% (1+)	
25	kidney tumor	f	3Y8M	primary	nephroblastoma	kidney	y	blastemic type	under therapy	High risk	n.d.		15% nuclear (1+)
26	kidney tumor	m	7Y 2M	primary	nephroblastoma	kidney	y	blastemic type	follow up	High Risk	n.d.	0% (0+)	
27	kidney tumor	f	11Y7M	primary	nephroblastoma	kidney	y	epithelial type St 1	follow up	Intermediate	5,94E+02	0% (0+)	
28	kidney tumor	f	1Y10M	primary	nephroblastoma	kidney	y	stroma type	follow up	Intermediate	2,79E+04	n.d.	
28	kidney tumor	f	1Y10M	primary	nephroblastoma	kidney	y	stroma type	follow up	Intermediate	4,60E+04	n.d.	
29	kidney tumor	f	6Y 2M	primary	nephroblastoma	kidney	y	stroma type	follow up	Intermediate	n.d.	5% (1,5+)	
30	kidney tumor	f	5Y3M	metastasis	nephroblastoma	kidney	y	mixed type	follow up	Intermediate	2,78E+03	n.d.	
30	kidney tumor	f	4Y9M	primary	nephroblastoma	kidney	y	mixed type	follow up	Intermediate	1,27E+03	0% (0+)	
30	kidney tumor	f	7Y0M	metastasis	nephroblastoma	liver	y	mixed type	follow up	Intermediate	3,06E+03	n.d.	50% nuclear (1+)
30	kidney tumor	f	7Y0M	metastasis	nephroblastoma	liver	y	mixed type	follow up	Intermediate	3,29E+03	n.d.	
31	kidney tumor	m	3Y7M	primary	nephroblastoma	kidney bilateral	y	mixed type	follow up	Intermediate	1,01E+03	0% (0+)	
32	kidney tumor	f	2Y1M	primary	nephroblastoma	kidney	y	mixed type	follow up	Intermediate	1,96E+05	n.d.	
33	kidney tumor	m	1Y6M	primary	nephroblastoma	kidney	y	mixed type	follow up	Intermediate	5,66E+03	n.d.	
34	kidney tumor	f	2Y9M	primary	nephroblastoma	kidney	y	mixed type St 2	follow up	Intermediate	7,27E+04	25% (2+)	
35	kidney tumor	f	9Y10M	primary	nephroblastoma	kidney bilateral	y	mixed type St 4	follow up	n.d	1,82E+03	0% (0+)	
36	kidney tumor	m	3Y4M	primary	nephroblastoma	kidney	n	mixed type	follow up	Intermediate	2,29E+04	n.d.	
36	kidney tumor	m	3Y4M	primary	nephroblastomatosis	kidney	y	mixed type	follow up	Intermediate	4,61E+03	50% of the immature epithelial	
